# Mental Health and Behavior of College Students During the COVID-19 Pandemic: Longitudinal Mobile Smartphone and Ecological Momentary Assessment Study, Part II

**DOI:** 10.2196/28892

**Published:** 2021-06-04

**Authors:** Dante L Mack, Alex W DaSilva, Courtney Rogers, Elin Hedlund, Eilis I Murphy, Vlado Vojdanovski, Jane Plomp, Weichen Wang, Subigya K Nepal, Paul E Holtzheimer, Dylan D Wagner, Nicholas C Jacobson, Meghan L Meyer, Andrew T Campbell, Jeremy F Huckins

**Affiliations:** 1 Department of Psychological and Brain Sciences Dartmouth College Hanover, NH United States; 2 Department of Computer Science Dartmouth College Hanover, NH United States; 3 National Center for PTSD White River Junction, VT United States; 4 Department of Psychiatry Dartmouth-Hitchcock Medical Center Lebanon, NH United States; 5 Department of Psychology Ohio State University Columbus, OH United States; 6 Department of Biomedical Data Science Geisel School of Medicine Dartmouth College Hanover, NH United States

**Keywords:** anxiety, college, COVID-19, COVID fatigue, depression, George Floyd, mobile sensing, phone usage, sleep, digital phenotyping

## Abstract

**Background:**

Since late 2019, the lives of people across the globe have been disrupted by COVID-19. Millions of people have become infected with the disease, while billions of people have been continually asked or required by local and national governments to change their behavioral patterns. Previous research on the COVID-19 pandemic suggests that it is associated with large-scale behavioral and mental health changes; however, few studies have been able to track these changes with frequent, near real-time sampling or compare these changes to previous years of data for the same individuals.

**Objective:**

By combining mobile phone sensing and self-reported mental health data in a cohort of college-aged students enrolled in a longitudinal study, we seek to understand the behavioral and mental health impacts associated with the COVID-19 pandemic, measured by interest across the United States in the search terms *coronavirus* and *COVID fatigue*.

**Methods:**

Behaviors such as the number of locations visited, distance traveled, duration of phone use, number of phone unlocks, sleep duration, and sedentary time were measured using the StudentLife mobile smartphone sensing app. Depression and anxiety were assessed using weekly self-reported ecological momentary assessments, including the Patient Health Questionnaire-4. The participants were 217 undergraduate students. Differences in behaviors and self-reported mental health collected during the Spring 2020 term, as compared to previous terms in the same cohort, were modeled using mixed linear models.

**Results:**

Linear mixed models demonstrated differences in phone use, sleep, sedentary time and number of locations visited associated with the COVID-19 pandemic. In further models, these behaviors were strongly associated with increased interest in *COVID fatigue*. When mental health metrics (eg, depression and anxiety) were added to the previous measures (week of term, number of locations visited, phone use, sedentary time), both anxiety and depression (*P*<.001) were significantly associated with interest in *COVID fatigue*. Notably, these behavioral and mental health changes are consistent with those observed around the initial implementation of COVID-19 lockdowns in the spring of 2020.

**Conclusions:**

In the initial lockdown phase of the COVID-19 pandemic, people spent more time on their phones, were more sedentary, visited fewer locations, and exhibited increased symptoms of anxiety and depression. As the pandemic persisted through the spring, people continued to exhibit very similar changes in both mental health and behaviors. Although these large-scale shifts in mental health and behaviors are unsurprising, understanding them is critical in disrupting the negative consequences to mental health during the ongoing pandemic.

## Introduction

### The COVID-19 Pandemic

COVID-19 has drastically changed life for people worldwide. The World Health Organization (WHO) declared COVID-19 a pandemic on March 11, 2020 [1]. In this work, we extend our previous research beyond the initial phases of the pandemic to June 25, 2020. By this time, the world had seen over 2,000,000 confirmed COVID-19 cases and 100,000 deaths [2], and the severe long-term political, economic, educational, and social ramifications of COVID-19 had grown increasingly acute. Understanding the behavioral and mental health implications for individuals during the spring of 2020, an unprecedented period of high stress, is critical for the upkeep of mental health during the pandemic.

College students seem to be vulnerable to the impact of COVID-19 on mental health [3-5]. In the spring of 2020, global health measures forced many colleges to shut down in an effort to mitigate the spread of the virus. On March 12, 2020, Dartmouth College requested that students leave the campus, with hope that the campus could resume normal operations in 5 weeks. At that time, it was believed that the COVID-19 situation would end rather quickly; however, by March 17, the severity of the virus became more apparent, and Dartmouth College announced that all classes and office hours would be held remotely during the Spring 2020 academic term. Students were asked to leave campus and not return until further notice. Following changes in school, local, and federal policies, drastic alterations in students’ behavior were observed. Prior research found that during the initial stages of the pandemic, this cohort of students visited fewer locations, spent more time sedentary, and increased its phone use [6]. All these behavioral changes were linked with increased levels of anxiety and depression [6], a trend observed by other researchers among various groups of college students [7,8]. Despite the negative impact on their mental health, many students continued to practice physical distancing with the hope of a swift return to normalcy. Unfortunately, improved knowledge about the ways in which COVID-19 spreads made it increasingly clear that physical distancing and other measures needed to continue for the sake of public health. Although some localities began to ease their restrictions and reopen in April 2020, it was still widely suggested that people avoid public gatherings and maintain physical distance from others.

### COVID-19 Restrictions Over the Long Term

**Figure 1:**
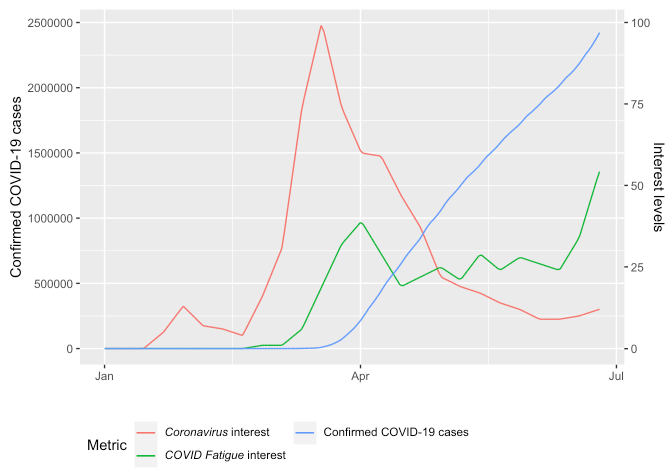
Number of confirmed COVID-19 cases graphed alongside search term interest (scaled from 0-100) during the winter and spring of 2020. COVID-19 case data were taken from the COVID-19 Data Repository by the Center for Systems Science and Engineering (CSSE) at Johns Hopkins University [2]. Search term interest was taken from Google Trends.

Towns and cities across the United States were forced to sustain COVID-19 “Stay Home, Stay Safe” restrictions in an effort to stem the spread of COVID-19. The effort of adhering to these restrictions seems to have bred fatigue among people nationwide. Although there is evidence that these stay-at-home-orders resulted in an immediate suppression of negative changes in mental health brought on during this time [9], people grew weary of the effort required to maintain constant vigilance. For many, staying at home began to feel like a monotonous rut—many referred to this feeling as “COVID fatigue.” Search term interest in *COVID fatigue* is seen alongside interest in *coronavirus* and the total number of confirmed COVID-19 cases [2] (Figure 1). Interest in *COVID fatigue* began to grow about a month into the pandemic (spiking in April) and continued to grow throughout the spring, while interest in *coronavirus* peaked around the time when policy changes were implemented in March before sharply and steadily declining. Therefore, *COVID fatigue* became an appropriate metric for measuring pandemic intensity at a given moment in time, while interest in the search term *coronavirus* seemed to capture the initial pandemic-related changes, reaching peak interest levels in late March. because many students continued to live under “Stay Home, Stay Safe” restrictions throughout the spring, it is necessary to understand how the sustenance of these behaviors is related to mental health.

Over the last year, the COVID-19 pandemic has prompted multiple lockdowns, which have brought about increased interest in the search term *COVID fatigue*, a term that has quickly become the subject of much discussion in public health research [10-13]. Cross-sectional research in Istanbul, Turkey, suggests that those with higher education levels experience more COVID-19–based fatigue [14], which may be attributable to a greater likelihood of this population to adhere to virus protocols and practice physical distancing [14,15]. Recent work proposes that college students may experience increases in depression due to COVID fatigue [16]. Most work measuring COVID fatigue has been limited to the use of web-based surveys. Using smartphone mobile sensing for digital phenotyping enables increased sampling frequency [17] and an ecologically valid approach [18,19], particularly when paired with ecological momentary assessments (EMAs). How behavioral and mental health changes related to COVID fatigue among populations at high risk for mental health issues (eg, college students) remains an unresolved question that we investigate in this work.

### Modeling Stages of the Pandemic With Changes in Behavior and Mental Health

Using previously successful methods, this work seeks to infer the behavioral and mental health impacts of the initial onset and duration of the COVID-19 pandemic among a cohort of Dartmouth College undergraduate students. Search query–related data are a promising metric in gathering population-based interest, which can be used to successfully identify changes in mental health [9,20,21]. Prior work has shown EMAs to be an excellent tool for gathering real-time assessments of mental health [22]. Notably, researchers have also used EMAs to assess mental health changes during the COVID-19 pandemic [6,23]. Interest in the search terms *coronavirus* and *COVID fatigue* was gathered from Google Trends to quantify the initial and ongoing phases of the pandemic (Figure 1). Interest levels were linearly inferred based on smartphone behavioral sensing data (gathered via passive sensing) and reported levels of anxiety and depression. Linear models are a useful tool in understanding the impact of the COVID-19 pandemic on daily behaviors and mental health in previous work, as a nearly identical method using COVID-19 media presence was previously successful [6]. Previously, we observed that the early media presence of COVID-19 was associated with increased levels of anxiety and depression as well as increased sedentary time and phone use [6]. This work seeks to build on prior work conducted by this and other groups to gain further insight into the ways in which COVID-19 has impacted daily life and mental health. We hypothesized that over nearly 3 months, students would continue to exhibit changes in depression, anxiety, and daily behaviors similar to those seen in the initial response to the COVID-19 pandemic.

### Objective

In this work, we seek to understand the behaviors and mental health changes associated with the continuing phases of the pandemic, as inferred by interest in the search terms *COVID fatigue* and *coronavirus*.

## Methods

### Study Design

All data from this study were obtained from the StudentLife study [24], which is a longitudinal multimodal study designed to follow the experiences of undergraduate students throughout their academic tenure with a focus on mental health. Study components include smartphone mobile sensing through the StudentLife app [25], EMAs, and surveys focusing on a variety of college experience components and functional neuroimaging [26].

### Participants

Data were collected from 219 participants who agreed to provide mobile sensing data via the StudentLife app [25]. One participant was removed from the study for having a phone incompatible with the app, and one participant withdrew within a week of starting the study. Data for both participants were excluded from further analyses. Of the remaining 217 participants, 67.8% (147) were female, with an age range of 18-22 years at the time of enrollment. Recruitment for this study began in August 2017 and concluded in November 2018. This study was reviewed and approved by Dartmouth College’s Committee for the Protection of Human Subjects. All data analyzed were collected from September 9, 2017, through June 25, 2020.

### Academic Terms

At Dartmouth College, the academic schedule consists of a flexible, year-round calendar that is roughly divided into 4 10-week academic terms (quarters), typically followed by 2 or more weeks of break. COVID-19 was first discovered in the United States during Dartmouth College’s Winter 2020 term, which began on January 6, 2020. The Spring 2020 academic term (March 30 start date) was the first complete academic term that occurred during the COVID-19 pandemic. To graphically show differences in anxiety and depression related to COVID-19, academic terms, including the subsequent 2 weeks of break prior to the Winter 2020 term, were included as control terms.

### Mobile Sensing and Ecological Momentary Assessments

Smartphone sensing data and EMA surveys were administered using the StudentLife application (iOS and Android) [25]. The StudentLife app collects data from several of the phone’s sensors, including but not limited to GPS, accelerometer, and lock/unlock status. Anonymized data from the StudentLife app are uploaded to a secure server whenever a participant is both connected to a Wi-Fi network and charging their phone. Data from these sensors are used to assess items such as the day-to-day and week-by-week impact of workload on stress, sleep, activity, mood, sociability, mental well-being, and academic performance [25]. Students are prompted weekly by the StudentLife application to complete a few short surveys, administered as EMAs [22]. These EMAs include the Patient Health Questionnaire-4 (PHQ-4), which combines a brief measure of depressive and anxious symptoms [27] that assesses how often individuals were bothered by specific symptoms over the last 2 weeks with values ranging from 0 to 6 for each subscale. The PHQ-4 combines the Patient Health Questionnaire-2 (PHQ-2) and the Generalized Anxiety Disorder-2 (GAD-2) tool.

#### Sedentary Time

Sedentary time, or stationary duration, is computed with algorithms that detect a lack of movement based on accelerometer data from a phone’s sensors. This enables us to measure students’ activity or, more precisely, their lack of activity level. The StudentLife app continuously infers physical activities using the Android activity recognition application programming interface [28,29] or iOS Core Motion [30].

#### Sleep

Sleep was inferred through a combination of passive sensing features (ambient light, movement activity, screen on/off). In this way, three features were computed: sleep onset, wake time, and sleep duration. These measures of sleep have been shown to be accurate within ±30 minutes of total sleep duration [25].

#### Location

Density-based spatial clustering of applications with noise (DBSCAN) [31] was used to cluster GPS coordinates to determine the number of locations visited and distance traveled during a given time period. Locations were detected when 3 GPS samples (sampled every 10 minutes) were within a radius of 30 meters. Distance was calculated in meters traveled between all locations throughout the day.

#### Phone Use

Unlock duration is a measurement of time during which a user’s phone is unlocked and the screen is on, calculated as the time between the user unlocking the phone and the user either manually relocking the phone or autolocking due to disuse (the iOS default is 30 seconds, Android defaults vary by manufacturer, and users can alter this time through their phone settings). Notification and system services do not influence the measurement of unlock duration. For the iOS app (189 users), from the start of the study in September 2017 until September 2018, unlock duration was measured by remotely triggering phones every 10 minutes, sampling 1 minute every 10 minute period (minimum 10% temporal coverage). After September 2018, phones were remotely triggered every 3 minutes, with subsequent sampling for 1 minute. Lock/unlock behaviors within that minute are recorded in real time, while locks/unlocks for the remaining 2 minutes are logged during the next remote trigger. The Android phone app natively supports phone use tracking and does not need to be triggered (28 users).

### Google Trends

Google is the world’s leading search engine, and Google Trends is an excellent tool to quantify topic interest. In turn, interest data from Google Trends have been successfully used to quantify changes in mental health [9,20,21]. Google data are posted publicly, and data can be downloaded directly through Google’s web portals or through freely available software. Google normalizes search data by region (in this case, the United States). To normalize the data, each data point is divided by the total searches of the geographic and time range it represents to compare relative popularity; the results are then scaled from a range of 0 to 100 based on a topic’s proportion to all searches on all topics [32]. Search terms with low interest appear as “0,” while “100” reflects peak interest during a given period. To obtain an unbiased measurement of interest in *coronavirus* and *COVID fatigue*, interest in both search terms was pulled for the duration of the entire study (September 7, 2017, to June 25, 2020). Data received from Google Trends were reported on a weekly scale; interest was reported for each Sunday. To gather a more finely grained real-time view of interest, the data was linearly interpolated using the “approxm” function from the Freqprof package in RStudio.

### Data Processing, Modeling, and Visualization in RStudio

**Figure 2:**
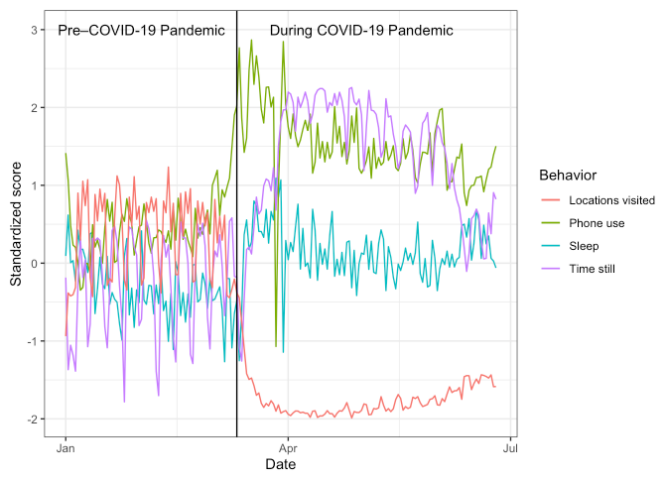
Standardized daily scores of the number of locations visited, duration of time phone unlocked, sleep duration, and sedentary time before and after the World Health Organization declared COVID-19 a global pandemic (March 11, 2020). All behaviors were calculated using data from the StudentLife app.

Modeling was implemented in lme4 [33] and lmerTest [34]. All plots were generated using ggplot2 [35]. Result tables were produced using stargazer [36]. On average, 164/217 participants (75.6%) reported mobile sensing data each day. For all participants with data, for the following behaviors, inferred through mobile sensing, daily group-level averages were calculated, standardized, and plotted over the time course of January to July 2020: locations visited, phone use, sleep, and time still (Figure 2). The objective of the analyses was to determine how the shock and sustained nature of the pandemic impacted the participants’ behaviors and mental health. The term week was modeled as linear and quadratic factors. To obtain a variable mirroring interest in *coronavirus* and *COVID fatigue*, topic interest in both *coronavirus* and *COVID fatigue* was gathered from Google Trends. Interest levels were modeled with fixed effects of reported depression and anxiety levels, unlock duration, unlock number, sedentary time, sleep duration, number of locations visited, and term week (linear and quadratic) variables, as well as random intercepts for each participant. For each variable, any days with missing data (14.47%) were excluded from the analysis. Each variable except participant was scaled to aid model convergence. *P* values were calculated using the Satterthwaite method as implemented in lmerModLmerTest as part of lmerTest.

## Results

### Behavioral and Mental Health Changes Associated With the COVID-19 Pandemic

**Figure 3:**
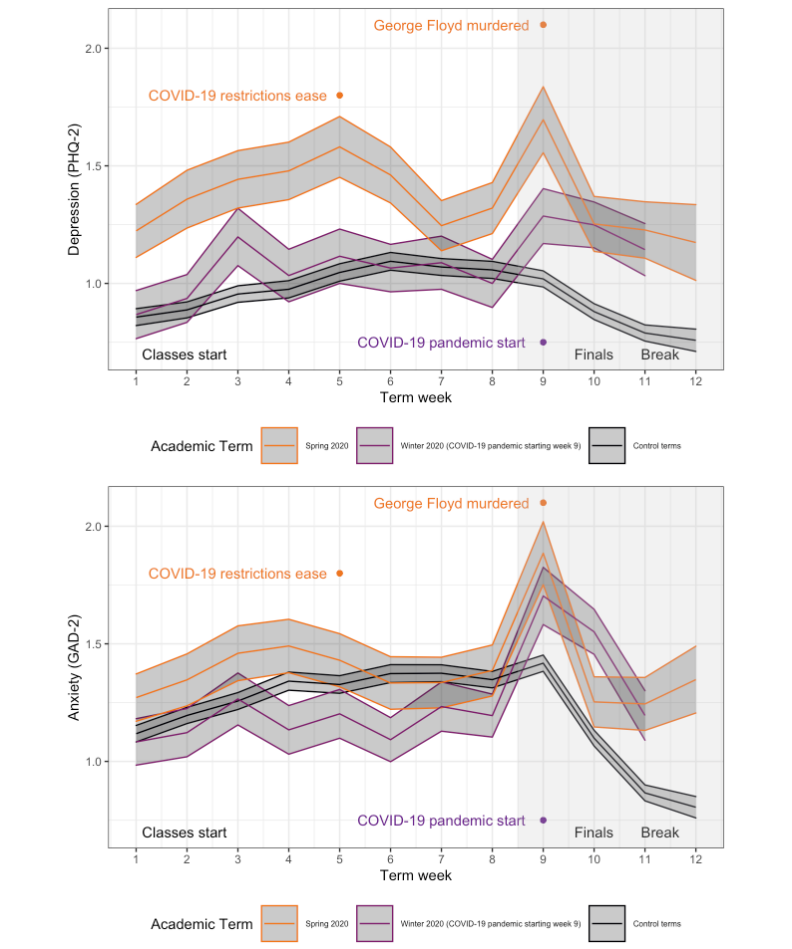
Depression (top) and anxiety (bottom) scores across an academic term and the first 2 weeks of break, with the term influenced by the outbreak of the COVID-19 pandemic as a separate line. The shaded ribbons represent the standard error for each week. Winter 2020 weeks influenced by policy changes related to COVID-19 are represented with a shaded box from weeks 9 to 12. Colored text corresponds to the term in which the event took place. Depression and anxiety were measured with the PHQ-2 and GAD-2 scales through the StudentLife app. Control terms include data from the same group of individuals across previous academic terms. Finals: final examinations; GAD-2: Generalized Anxiety Disorder-2; PHQ-2: Patient Health Questionnaire-2.

To visually observe shifts in behaviors due to the COVID-19 pandemic, standardized scores were produced for each behavior plotted with a vertical line marking the date on which the WHO named COVID-19 a pandemic (Figure 2). Students visited fewer locations, used their phones more, spent more time sedentary, and initially exhibited increases in sleep before a sustained decrease in sleep. Notably, the graph shows less extreme peaks and valleys, suggesting that differences in behavior between weekdays and weekends were less pronounced during the COVID-19 pandemic. The mean values of depression and anxiety were plotted by week of the term; data were combined from all study participants from all terms prior to the Winter 2020 term, which was plotted as a separate line along with the Spring 2020 term. Standard error was plotted as a shaded ribbon surrounding the mean. Sustained increased levels of depression and anxiety throughout the Spring 2020 academic term compared to Winter 2020 and control terms were plotted (Figure 3). Peak self-reported anxiety and depression symptoms were observed during the ninth week of the Spring term, corresponding to the time before final examinations, increases in COVID-19 cases, and the murder of George Floyd. Linear models were produced to better assess these differences in a more quantitative manner.

Multiple models for sedentary time, phone use, sleep, locations visited, depression, and anxiety were tested (see the *Methods* section for specific details). For all mobile sensing variables except phone use, superior fit was observed with the most complex model that included the COVID-19 academic term, linear and quadratic term week trends, the interaction of the COVID-19 academic term with each of the term week trends, and allowance for random intercepts for each participant’s data (random effects). For phone use, superior fit (measured by lowest deviance) was observed with the model including the COVID-19 academic term, linear and quadratic term week trends, and the interaction between the linear term week variable and the COVID-19 academic term. Modeling of the COVID-19 academic term compared with non–COVID-19 academic terms identified significantly increased sedentary time, phone use, sleep, depression, and anxiety, along with a decrease in locations visited (*P*<.001; Table 1). Significant interactions of the COVID-19 term and quadratic term week regressor for sedentary time, locations visited, and anxiety (*P*<.001), as well as sleep and depression (*P*=.05), were observed. Significant interactions between the COVID-19 term and the linear term week regressor for locations visited, phone use, and anxiety (*P*<.001) as well as sedentary time (*P*=.01) and depression (*P*=.05) were also observed.

**Table 1:** Models of sedentary time, locations visited, sleep duration, phone use, depression, and anxiety by week and by presence of COVID-19 during the academic term.

Variable	Sedentary time (1) (91,541 observations)		Locations visited (2) (85,148 observations)				Sleep duration (3) (91,541 observations)			Phone use (4) (91,541 observations)			Depression (5) (21,284 observations)			Anxiety (6) (21,284 observations)	
	Standardized coefficient (SE)	*P* value		Standardized coefficient (SE)	*P* value	Standardized coefficient (SE)		*P* value	Standardized coefficient (SE)		*P* value	Standardized coefficient (SE)		*P* value	Standardized coefficient (SE)		*P* value
COVID-19 Term	0.469 (0.007)	<.001		–0.851 (0.008)	<.001	0.061 (0.008)		<.001	0.406 (0.007)		<.001	0.262 (0.013)		<.001	0.150 (0.013)		<.001
Term week (linear)	–0.056 (0.003)	<.001		–0.218 (0.003)	<.001	0.041 (0.003)		<.001	0.072 (0.003)		<.001	–0.014 (0.006)		.02	–0.042 (0.006)		<.001
Term week (quadratic)	–0.036 (0.003)	<.001		–0.140 (0.003)	<.001	0.060 (0.003)		<.001	0.013 (0.003)		<.001	–0.074 (0.006)		<.001	–0.108 (0.006)		<.001
COVID-19 term: term week (linear)	–0.021 (0.007)	.003		0.121 (0.008)	<.001	–0.005 (0.007)		.50	–0.032 (0.006)		<.001	0.033 (0.013)		.01	0.086 (0.013)		<.001
COVID-19 term: term week (quadratic)	0.025 (0.007)	.001		0.071 (0.008)	<.001	–0.017 (0.007)		.02	N/A^a^		N/A	0.029 (0.013)		.02	0.088 (0.013)		<.001
Constant	–0.099 (0.033)	.003		0.148 (0.025)	<.001	0.010 (0.032)		.75	–0.102 (0.043)		.02	–0.011 (0.046)		.82	0.031 (0.045)		.49

^a^N/A: not applicable.

### COVID-19 Interest, Mental Health, and Mobile Sensing

After observing broad differences in sleep, phone use, locations visited, sedentary time, depression, and anxiety between the Winter 2020 term and previous terms, the next goal was to see if these behaviors changed on a daily scale, particularly mirroring the relative interests in *coronavirus* and *COVID fatigue* over the duration of the study. Interest in both variables was at zero until March 2020 (Figure 1). To gather what behaviors changed with *coronavirus* interest and how they changed, we included fixed effects for phone use (unlock duration and unlock number), sedentary time, sleep duration, number of locations visited, and linear and quadratic academic term week regressors. Random intercepts per participant were included in the model. Each variable was scaled to help with convergence of the restricted maximum likelihood model and to obtain regression coefficients that can be compared for relative importance. Models inferring interest in *COVID fatigue* had a better fit (lower deviance) than models of *coronavirus* interest. All variables were significantly associated with interest in the search term *coronavirus*, and all variables except distance traveled were significantly associated with interest in *COVID fatigue*. In both models, phone use (unlock duration) had the largest positive standardized coefficient, followed by sedentary time. The number of locations visited had the largest magnitude of negative standardized coefficient, followed by the number of phone unlocks and sleep duration (*P*<.001).

**Figure 4:**
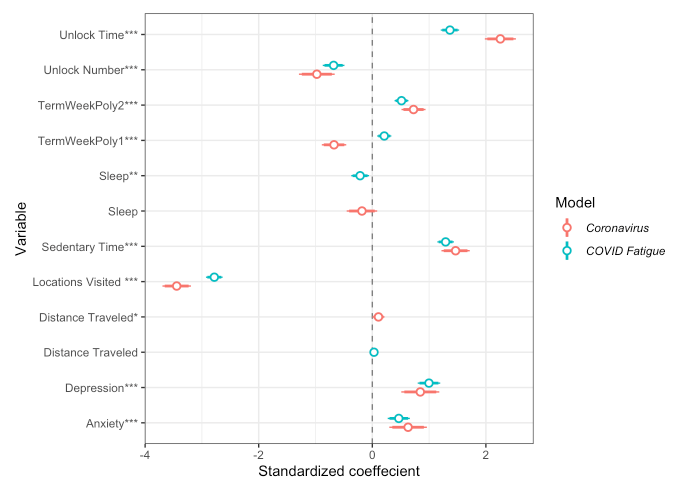
Coefficient plots from mixed linear models of interest in the search terms *coronavirus* and *COVID fatigue*, inferred by mobile smartphone features and self-reported mental health. Intercepts and random intercepts per participant are not plotted. *P<.05; **P<.01; ***P<.001.

In the second set of models of interest data, we again made inferences with mobile sensing features, this time with the addition of self-reported anxiety and depression scores. Again, all variables seemed to produce a better fit when inferring interest in COVID fatigue. When anxiety and depression were added to the previously used sensing model, our model demonstrated that increased anxiety and depression were significantly associated with rising interest in *coronavirus* and *COVID fatigue* (*P*<.001). Again, we observed increased phone use (unlock duration), increased sedentary time, and decreased number of locations visited, with standardized beta weights stable across both models. Sleep was negatively associated with interest in *COVID fatigue* (*P*=.05) but no longer had a significant association with *coronavirus* interest, perhaps due to the weekly measured mental health metrics accounting for variance. Notably, both the linear and quadratic term week variables were positively associated with *COVID fatigue* interest, though only the quadratic term week variables were positively associated with *coronavirus* interest. A coefficient plot depicting the relative impacts on behaviors and mental health is shown in Figure 4.

## Discussion

### Principal Findings

As in our previous work, we found increased interest in COVID-19 to be associated with increased depression and anxiety alongside stark behavioral changes. Further linear models showed that as initial interest in the search term *coronavirus* increased, a cohort of 217 Dartmouth College undergraduate students became more sedentary, visited fewer locations, slept less, used their phones more, and showed increases in anxiety and depression. Although initial models suggested an increase in sleep during academic terms affected by COVID-19, controlling for other sensing variables revealed that students exhibited decreases in sleep as the pandemic endured. Although we observed an increase in distance traveled as *coronavirus* interest rose, we attribute this finding to students returning home, as it coincides with Dartmouth College closing its campus. We found that the search term *coronavirus* had a stronger relationship with changes in behaviors and mental health, with the exception of levels of depression. With sustained adherence to “Stay Home and Stay Safe” guidelines implemented by many national and international governments, we found that as the pandemic endured, students were more sedentary, visited fewer locations (as inferred from GPS tracking), slept less, used their phones more, and exhibited increases in anxiety and depression. Notably, over the entire course of the study, self-reported anxiety and depression levels were highest in the week after George Floyd was murdered. Future digital phenotyping work should investigate the impact of current events on mental health and behavior.

Our models revealed that behaviors and mental health changed in response to the pandemic over a sustained period of time. Although this work is descriptive by nature, behavioral changes sometimes drive changes in mental health in a lagged manner. Notably, a comparison of contemporaneous correlations of data collected prior to and during the COVID-19 pandemic revealed a decrease in the strength of the correlations between sedentary time and mental health (anxiety and depression). Notably, the correlation between locations visited and anxiety also became weaker. Future COVID-19–related work investigating causal relationships between general daily travel, particular sedentary behaviors, and changes in mental health would be of high interest.

Unfortunately, we remain engaged in a battle against the continued spread of COVID-19, necessitating long-term adherence to physical distancing and other mitigation protocols. Although the initial impact of these unprecedented shifts in behavior has been documented, the effects of the longevity of the pandemic are less known. This work uses longitudinal data from a cohort of 217 college students to observe what behavioral and mental health changes are associated with the enduring pandemic. As in previous work, we found increased interest in COVID-19 to be associated with increased depression and behavioral changes. Further linear models showed that as initial interest in *coronavirus* increased, participants became more sedentary, visited fewer locations, slept less, used their phones more, and showed increases in anxiety and depression. Although initial models suggested an increase in sleep during academic terms affected by the COVID-19 pandemic, controlling for other sensing variables revealed that students exhibited decreases in sleep as the pandemic endured.

As in previous work [6], we again observed that COVID-19 is associated with decreased sleep, increased sedentary time, and negative mental health outcomes. Recent work, collected prepandemic, suggests that sleep may mediate a relationship between physical activity and stress [37]. An overwhelming body of research supports the claim that increased physical activity is associated with longer, better quality sleep [38-40]. On the other hand, physical inactivity tends to be associated with increased stress [41], and recent work suggests that exercising during the pandemic may help reduce levels of stress [42]. Stress has been implicated in changes in mental health [43,44] and poor sleep [45-47]. Importantly, other work identifies disturbances in sleep and physical activity that coincide with COVID-19 fatigue [12]. Although the observed prolonged decreases in sleep may potentially mediate a relationship between sustained decreased physical activity and mental well-being during the pandemic, mental health research currently remains uncertain.

Our finding of sustained increased phone use during the COVID-19 pandemic presents a quandary. Work prior to the pandemic suggests that smartphone overuse is associated with negative changes in mental health [48]. However, at a time of widespread social distancing, it seems that smartphones may have a more complex relationship with mental health. Recent research has shown that using smartphones to connect with friends and colleagues may be associated with increased well-being during the pandemic [49]. Moreover, social connection may provide resilience in battling COVID-19 fatigue [50]. Simultaneously, increased phone use during the pandemic may reflect increased social media use and negative changes in mental health [49,51]. Given the observance of increased phone use among college students during the COVID-19 pandemic, future work may seek to uncover the various impacts of phone use during the pandemic.

Primary takeaways from the models are that individuals are maintaining previously seen changes in behavior and mental health. This finding suggests the importance of prioritizing communication with loved ones, and it emphasizes the benefit of maintaining a regular sleep schedule and physical activity to help improve mental health.

### Study Limitations and Future Directions

One possible limitation of this study is the search terms used. Although other literature reports use the term “COVID fatigue” to refer to behavioral exhaustion, it is worth noting that similar search terms listed on Google Trends are related to the symptomatology of COVID-19 (ie, fatigue related to virus contraction) rather than to behavioral fatigue. Our graphs show that interest in *COVID fatigue* begins to increase well into the pandemic, but after interest in *coronavirus* increases; however, it is possible that we are partially capturing interest in symptoms. Still, we observed that behaviors and mental health model interest in *covid fatigue* with a better fit than with interest in the term *coronavirus*; this finding suggests that the gradually building interest in COVID-19 fatigue accurately reflects the continuing phases, while interest in the disease itself reflects the initial phase. Future work may consider identifying trending search terms such as *covid mutation* or *covid vaccine* to help quantify the interest in the continuation of the pandemic and how that interest may be impacting mental health. Similarly, as vaccines are more widely distributed, using the terms *covid vaccine* and *herd immunity* along with the terms used in this work could be an effective way to model the impacts of later and (hopefully) final stages of the COVID-19 pandemic on mental health.

Although the mobile smartphone sensing data gathered by the StudentLife app are quite robust, they too have potential limitations. When mobility is decreased, such as during a stay-at-home order, people may not always have their phones with them, which could lead to an overestimation of sedentary time and sleep. Our moderate sample size of 217 total participants across all terms and 212 participants during the COVID-19 pandemic (97.6%) limits the generalizability of the findings. Further, participants may be preferentially accessing larger screens (eg, tablets, laptops, or televisions). Because the app was only installed on the students’ mobile phones, our metric of phone use (as measured by screen unlock duration or number of unlocks) may underestimate the total amount of screen time. However, we did observe increased phone use, suggesting that the effect sizes we observe are underestimated. Future digital phenotyping work capable of distinguishing different types of screen time during the pandemic would be of high interest, as would work that distinguishes the purposes (ie, working, watching Netflix, reading) and proportions of screen use. Further work could also use smartwatches to improve the measurement of behaviors such as sedentary time and allow for more frequent sampling of phone use, location and other measures. Additionally, the use of smartwatches could facilitate highly accurate inferences of time spent exercising, sleep and sedentary time, as these watches typically track heart rate. Despite the moderate sample size and potential data inaccuracies, strong significant effects on mental health and behaviors were observed, suggesting robust effects.

Although finding ways to combat COVID-19 fatigue is beyond the scope of this work, we encourage more research on the subject. In our work, we were unable to infer daily changes in mental health, as to maximize long-term retention, the StudentLife app prompted participants to respond to weekly, not daily, EMAs. Although we presume that college students are likely to be more susceptible to experiencing pandemic fatigue, we do not directly gather fatigue-related feedback. Future studies may consider addressing fatigue more directly, although one should be cautious when choosing metrics of fatigue, as they may also be symptoms of depression (eg, physical exhaustion, anhedonia, trouble sleeping). Administering EMAs on a daily, rather than weekly, basis could help parse day-to-day changes in mental health related to fatigue. In light of the drastic changes in sedentary time, it is critical to find day-to-day activities or routines that may help prevent or reduce fatigue levels. There may be differences in the degree of reported mental health changes between those who spend more time watching television compared to those using social media. Although gathering enough participants may be challenging, using questionnaires to investigate specific behaviors that drive or reduce fatigue levels is critical.

### Comparison With Prior Work

In the initial stage of the COVID-19 pandemic, as the media presence of COVID-19 increased, individuals exhibited increased levels of depression and anxiety alongside increased sedentary time, phone use, and a decrease in the number of locations visited. This work examined if the same cohort of individuals exhibited the same changes in behaviors and mental health over the course of a 12-week academic term. Rather than using media presence, this work uses search term interest gathered from Google Trends, a tactic that has recently proved fruitful during the pandemic [9,20]. In this work, we chose the search term *COVID fatigue* to measure the intensity of the pandemic at a given moment to add to the prior knowledge gained during the initial phases of the pandemic. This tactic enabled us to observe what behavioral and mental health changes were associated with the ongoing COVID-19 pandemic, as opposed to focusing on the initial phase, as in prior work. In addition to previously observed shifts in mental health and behaviors, we observed significant decreases in sleep as the COVID-19 pandemic endured.

### Conclusions

While the long fight against COVID-19 continues, we must further our understanding of its impacts on mental health. This study provides additional insight into mental health and related behaviors during the ongoing COVID-19 pandemic. Using mixed linear models of smartphone mobile sensing and self-reported mental health questions, we were able to infer the initial and ongoing phases of the COVID-19 pandemic, as measured by search term interest in *coronavirus* and *COVID fatigue*; moreover, we were able to validate that the participants’ mental health and related behaviors changed contemporaneously with relative interest levels in these terms. Increases in depression, anxiety, sedentary time, and phone use, alongside decreases in sleep and the number of locations visited, are significantly associated with the ongoing pandemic. All of these changes seem very similar to those seen during the initial phases of the pandemic. As the virus continues to mutate, in future work, we may attempt to identify behaviors as resilience factors for changes in mental health. Similarly, work tracking the distribution of vaccines may elucidate a return to pre–COVID-19 pandemic levels of behavior and mental health.

## Abbreviations

DBSCAN: density-based spatial clustering of applications with noise
EMA: ecological momentary assessment
GAD-2: Generalized Anxiety Disorder-2
PHQ-2: Patient Health Questionnaire-2
PHQ-4: Patient Health Questionnaire-4
WHO: World Health Organization

## References

[1] Cucinotta D, Vanelli M (2020). WHO declares COVID-19 a pandemic. Acta Biomed.

[2] Dong E, Du H, Gardner L (2020). An interactive web-based dashboard to track COVID-19 in real time. Lancet Infect Dis.

[3] Cao W, Fang Z, Hou G, Han M, Xu X, Dong J, Zheng J (2020). The psychological impact of the COVID-19 epidemic on college students in China. Psychiatry Res.

[4] Hasan N, Bao Y (2020). Impact of "e-Learning crack-up" perception on psychological distress among college students during COVID-19 pandemic: a mediating role of "fear of academic year loss". Child Youth Serv Rev.

[5] Zhang Y, Zhang H, Ma X, Di Q (2020). Mental health problems during the COVID-19 pandemics and the mitigation effects of exercise: a longitudinal study of college students in China. Int J Environ Res Public Health.

[6] Huckins J, daSilva Alex W, Wang W, Hedlund E, Rogers C, Nepal S, Wu J, Obuchi M, Murphy E, Meyer M, Wagner Dylan D, Holtzheimer Paul E, Campbell Andrew T (2020). Mental health and behavior of college students during the early phases of the COVID-19 pandemic: longitudinal smartphone and ecological momentary assessment study. J Med Internet Res.

[7] Wang Z, Yang H, Yang Y, Liu D, Li Z, Zhang X, Zhang Y, Shen D, Chen Pei-Liang, Song Wei-Qi, Wang Xiao-Meng, Wu Xian-Bo, Yang Xing-Fen, Mao Chen (2020). Prevalence of anxiety and depression symptom, and the demands for psychological knowledge and interventions in college students during COVID-19 epidemic: A large cross-sectional study. J Affect Disord.

[8] Liu X, Liu J, Zhong X Psychological state of college students during COVID-19 epidemic. SSRN Journal..

[9] Jacobson NC, Lekkas D, Price G, Heinz MV, Song M, O'Malley A James, Barr PJ (2020). Flattening the mental health curve: COVID-19 stay-at-home orders are associated with alterations in mental health search behavior in the United States. JMIR Ment Health.

[10] Harvey N (2020). Behavioral fatigue: real phenomenon, naïve construct, or policy contrivance?. Front Psychol.

[11] Bartoszek A, Walkowiak D, Bartoszek A, Kardas G (2020). Mental well-being (depression, loneliness, insomnia, daily life fatigue) during COVID-19 related home-confinement-a study from Poland. Int J Environ Res Public Health.

[12] Field T, Mines S, Poling S, Diego M, Bendell D, Veazey C (2021). COVID-19 lockdown fatigue. American Journal of Psychiatric Research and Reviews.

[13] Rahmandad H, Lim TY, Sterman J (2021). Behavioral dynamics of COVID-19: estimating underreporting, multiple waves, and adherence fatigue across 92 nations. Syst Dyn Rev.

[14] Morgul E, Bener A, Atak M, Akyel S, Aktaş S, Bhugra D, Ventriglio A, Jordan TR (2020). COVID-19 pandemic and psychological fatigue in Turkey. Int J Soc Psychiatry.

[15] Carlucci L, D'Ambrosio Ines, Balsamo M (2020). Demographic and attitudinal factors of adherence to quarantine guidelines during COVID-19: the Italian model. Front Psychol.

[16] Ye B, Zhou X, Im H, Liu M, Wang X, Yang Q (2020). Epidemic rumination and resilience on college students' depressive symptoms during the COVID-19 pandemic: the mediating role of fatigue. Front Public Health.

[17] Faurholt-Jepsen M, Busk J, Þórarinsdóttir Helga, Frost M, Bardram J, Vinberg M, Kessing L (2019). Objective smartphone data as a potential diagnostic marker of bipolar disorder. Aust N Z J Psychiatry.

[18] Onnela J, Rauch SL (2016). Harnessing smartphone-based digital phenotyping to enhance behavioral and mental health. Neuropsychopharmacology.

[19] Insel T (2017). Digital phenotyping: technology for a new science of behavior. JAMA.

[20] Hoerger M, Alonzi S, Perry L, Voss H, Easwar S, Gerhart J (2020). Impact of the COVID-19 pandemic on mental health: real-time surveillance using Google Trends. Psychol Trauma.

[21] Tana J, Kettunen J, Eirola E, Paakkonen H (2018). Diurnal variations of depression-related health information seeking: case study in Finland using Google Trends data. JMIR Ment Health.

[22] Shiffman S, Stone AA, Hufford MR (2008). Ecological momentary assessment. Annu Rev Clin Psychol.

[23] Fried EI, Papanikolaou F, Epskamp S Mental health and social contact during the COVID-19 pandemic: an ecological momentary assessment study. PsyArXiv..

[24] StudentLife study. Dartmouth College.

[25] Wang R, Chen F, Chen Z, Li T, Harari G, Tignor S, Zhou X, Ben-Zeev D, Campbell A (2014). StudentLife: assessing mental health, academic performance and behavioral trends of college students using smartphones. UbiComp '14: Proceedings of the 2014 ACM International Joint Conference on Pervasive and Ubiquitous Computing.

[26] Huckins JF, daSilva AW, Wang R, Wang W, Hedlund EL, Murphy EI, Lopez RB, Rogers C, Holtzheimer PE, Kelley WM, Heatherton TF, Wagner DD, Haxby JV, Campbell AT (2019). Fusing mobile phone sensing and brain imaging to assess depression in college students. Front Neurosci.

[27] Kroenke K, Spitzer R, Williams J, Löwe B (2009). An ultra-brief screening scale for anxiety and depression: the PHQ–4. Psychosomatics.

[28] ActivityRecognitionApi. Google Play services.

[29] Wang R, Aung M, Abdullah S, Brian R, Campbell A, Choudhury T, Hauser M, Kane J, Merrill M, Scherer E (2016). CrossCheck: toward passive sensing and detection of mental health changes in people with schizophrenia. UbiComp '16: Proceedings of the 2016 ACM International Joint Conference on Pervasive and Ubiquitous Computing.

[30] IOS core motion. Apple Developer.

[31] Daszykowski M, Walczak B (2009). Density-based clustering methods. Comprehensive Chemometrics (Second Edition).

[32] Google Trends.

[33] Bates D, Mächler M, Bolker B, Walker S (2015). Fitting linear mixed-effects models Using lme4. J Stat Soft.

[34] Kuznetsova A, Brockhoff PB, Christensen RHB (2017). lmerTest package: tests in linear mixed effects models. J Stat Soft.

[35] Wickham H (2011). ggplot2. WIREs Comp Stat.

[36] Hlavac M (2018). Stargazer: well-formatted regression and summary statistics tables. R Project.

[37] Zhai X, Wu N, Koriyama S, Wang C, Shi M, Huang T, Wang K, Sawada SS, Fan X (2021). Mediating effect of perceived stress on the association between physical activity and sleep quality among Chinese college students. Int J Environ Res Public Health.

[38] Kredlow M, Capozzoli M, Hearon B, Calkins A, Otto M (2015). The effects of physical activity on sleep: a meta-analytic review. J Behav Med.

[39] Lang C, Brand S, Feldmeth AK, Holsboer-Trachsler E, Pühse Uwe, Gerber M (2013). Increased self-reported and objectively assessed physical activity predict sleep quality among adolescents. Physiol Behav.

[40] Loprinzi PD, Cardinal BJ (2011). Association between objectively-measured physical activity and sleep, NHANES 2005–2006. Ment Health Phys Act.

[41] Zhai X, Ye M, Wang C, Gu Q, Huang T, Wang K, Chen Z, Fan X (2020). Associations among physical activity and smartphone use with perceived stress and sleep quality of Chinese college students. Ment Health Phys Act.

[42] Deng C, Wang J, Zhu L, Liu H, Guo Y, Peng X, Shao J, Xia W (2020). Association of web-based physical education with mental health of college students in Wuhan during the COVID-19 outbreak: cross-sectional survey study. J Med Internet Res.

[43] van Praag H (2004). Can stress cause depression?. Prog Neuropsychopharmacol Biol Psychiatry.

[44] Huckins J, DaSilva A, Hedlund E, Murphy E, Rogers C, Wang W, Obuchi M, Holtzheimer P, Wagner D, Campbell A (2020). Causal factors of anxiety and depression in college students: longitudinal ecological momentary assessment and causal analysis using Peter and Clark momentary conditional independence. JMIR Ment Health.

[45] Caldwell K, Harrison M, Adams M, Quin RH, Greeson J (2010). Developing mindfulness in college students through movement-based courses: effects on self-regulatory self-efficacy, mood, stress, and sleep quality. J Am Coll Health.

[46] Lee S, Wuertz C, Rogers R, Chen Y (2013). Stress and sleep disturbances in female college students. Am J Hlth Behav.

[47] Amaral A, Soares M, Pinto A, Pereira A, Madeira N, Bos S, Marques M, Roque C, Macedo A (2018). Sleep difficulties in college students: the role of stress, affect and cognitive processes. Psychiatry Res.

[48] Demirci K, Akgönül Mehmet, Akpinar A (2015). Relationship of smartphone use severity with sleep quality, depression, and anxiety in university students. J Behav Addict.

[49] Sun R, Rieble C, Liu Y, Sauter D Connected despite lockdown: the role of social interactions and social media use in wellbeing. PsyArXiv..

[50] Nitschke J, Forbes P, Ali N, Cutler J, Apps M, Lockwood P, Lamm C (2021). Resilience during uncertainty? Greater social connectedness during COVID-19 lockdown is associated with reduced distress and fatigue. Br J Health Psychol.

[51] Gao J, Zheng P, Jia Y, Chen H, Mao Y, Chen S, Wang Y, Fu H, Dai J (2020). Mental health problems and social media exposure during COVID-19 outbreak. PLoS One.

